# Giant impact on early Ganymede and its subsequent reorientation

**DOI:** 10.1038/s41598-024-69914-2

**Published:** 2024-09-03

**Authors:** Naoyuki Hirata

**Affiliations:** Graduate School of Science, Kobe University, Rokkodai 1-1 657-8501, Kobe, Japan

**Keywords:** Planetary science, Rings and moons

## Abstract

Ganymede has an ancient impact structure called a furrow system. The furrow system is the largest impact structure in the outer solar system, and the impact should have significantly affected Ganymede’s early history; however, its effects are poorly understood. No attention has been given to the center of the furrow system coinciding with Ganymede's tidal axis, indicating that mass redistribution induced by the furrow-forming impact caused a reorientation (true polar wander) of Ganymede. We propose that the impact ejecta created a mass anomaly that reoriented the impact site toward the tidal axis. We found that an impactor with a radius of 150 km and an incidence angle between 60° and 90° most accurately reproduces the current location of the furrow system. We predict that future explorations would reveal remnant topographic profiles or gravity anomalies associated with the furrow-forming impact and reorientation. Additionally, various possible explanations for the reorientation of Ganymede, such as an impactor-origin mascon beneath the basin or a thickness variation in the lithosphere, should be studied.

## Introduction

**Figure 1:**
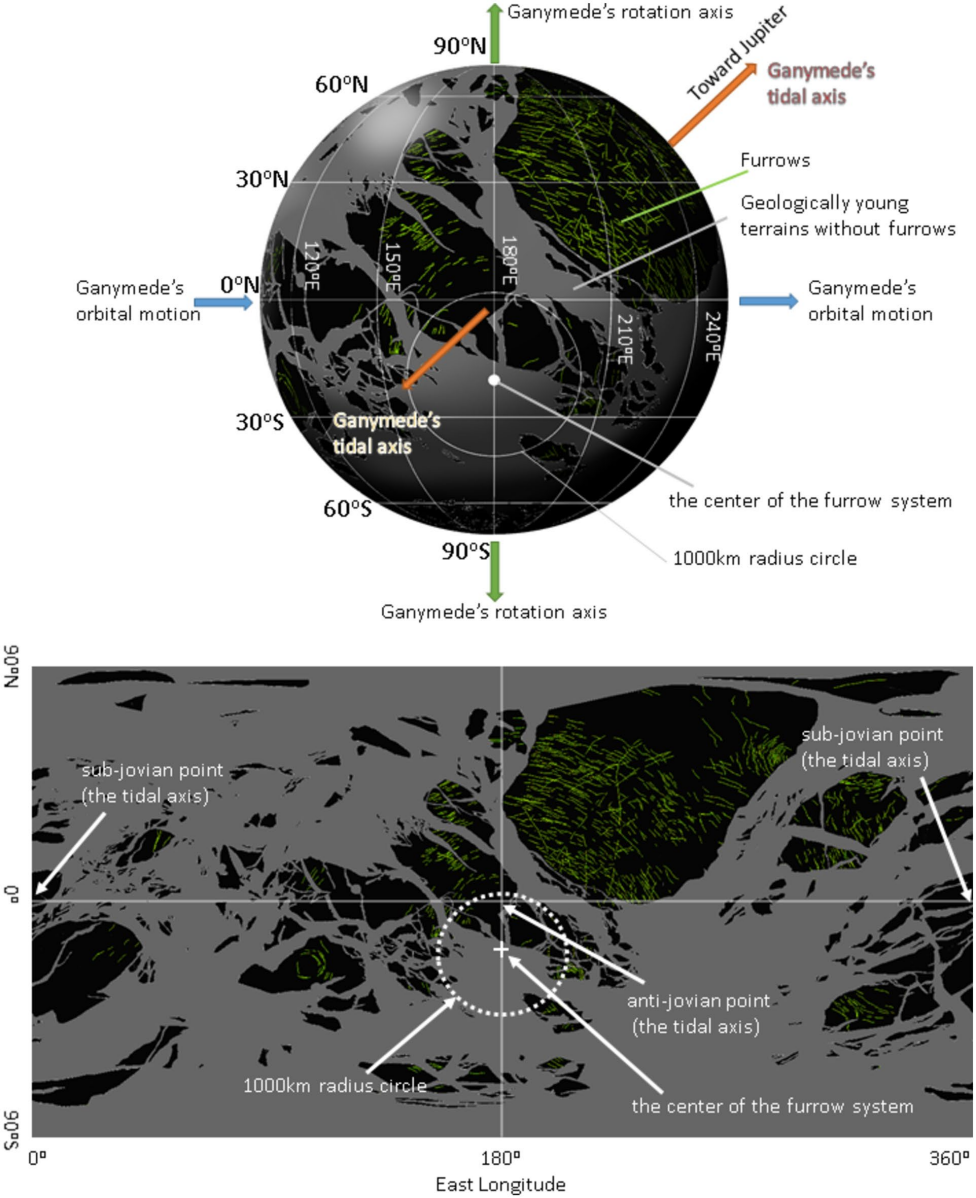
Distribution of furrows and location of the center of the furrow system shown in the hemisphere that always faces away from Jupiter (top) and the cylindrical projection map of Ganymede (bottom). The distribution of furrows was obtained from Collins et al.^10^. The gray regions represent geologically young terrain without furrows. Furrows (green lines) exist only on geologically old terrains (black regions).

Ganymede is the largest satellite in the solar system and has many unique features, including tectonic troughs known as furrows^1–5^. Furrows are the oldest surface features recognized on Ganymede because they are crosscut by any impact craters with diameters exceeding 10 km^3^. Therefore, furrows can provide a window into the early history of Ganymede. Furrows have been proposed to be fragments of multiring impact basin structures, similar to those of the Valhalla or Asgard basins on Callisto^1,2,6,7^. The largest furrow system is present across Galileo and Marius Regios (the so-called Galileo‒Marius furrow system), and it is the remnant of an ancient giant impact, which extends concentrically from a single point of Ganymede, 21° S 179° W^8^ (Fig. 1); however, estimating the size of the impactor is complicated because of the absence of an identifiable clear rim^8–11^. Although there are a few small furrow systems on Ganymede, only the Galileo–Marius furrow system is examined in this study.

We propose that the ejecta mass of the furrow-forming impact created a large positive gravity anomaly around the impact center, which subsequently led to the reorientation of Ganymede. No attention has been given to the fact that the center of the furrow system coincides with the longitude of Ganymede's tidal axis; however, this coincidence implies that Ganymede has experienced significant reorientation. Interestingly, the center of the furrow system has a very similar geometric location to that of the center of the Sputnik Planitia (18° N 178° E^12^), which is the largest impact basin on Pluto. Both centers are located along the tidal longitude and deviate poleward by 20° from the tidal axis. Note that the coordinates of Ganymede and Pluto are defined such that the tidal axes are at a latitude of 0° and longitudes of 0°/180°. Previous studies have proposed that the Sputnik Planitia has a large positive gravity anomaly that caused significant reorientation of Pluto^12–14^.

In general, a positive gravity anomaly on the surface of a tidally locked satellite leads to the reorientation of the satellite wherein the anomaly approaches the tidal axis; alternatively, a negative gravity anomaly leads to a reorientation wherein the anomaly approaches the rotation axis^15–18^. The crater hole and ejecta blanket act as negative and positive gravity anomalies, respectively. Both anomalies mostly cancel each other out, but the overall gravity anomaly becomes slightly negative, leading to a net effect of the reorientation of the crater center toward the rotation axis^19^. In fact, some of the largest impact basins, such as the South Pole Aitken basin (Moon) and Rheasilvia basin (Vesta), are located close to the polar region. In contrast, the Sputnik Planitia basin is located near the tidal axis. Previous studies^12,13^ have proposed that isostasy can be achieved within the basin floor if Pluto has a global ocean, which, together with the nitrogen deposits within the basin, creates a large positive gravity anomaly. Because the isostatically compensated topography (e.g., supported by the buoyancy of the asthenosphere or subsurface ocean) exhibits no free-air gravity anomaly, the negative gravity anomaly of the basin depression vanishes even if it has a negative topographic profile. In short, the basin depression of the Sputnik Planitia is likely to be compensated for and does not exhibit a negative gravity anomaly, whereas the ejecta blanket is not compensated for and thus exhibits a positive gravity anomaly.

A similar mechanism is appropriate for the case of the furrow-forming impact on Ganymede because the formation of multiring basins on the Jovian icy satellites has been explained by the collapse of a crater basin owing to fluid or warm convective ice beneath a thin ice shell^6,20–23^. Although it is difficult to argue whether Ganymede had a differentiated interior with an ocean or an undifferentiated interior without an ocean at the time of the formation of the furrow system, numerical simulations^24–27^ have shown that both a thin ice shell over the ocean and warm convective ice without an ocean can reproduce the shallowing of the basin floor and widening of the crater rim of the multiring basin. Isostasy would have been achieved within the basin floor in both subsurface models (see also Methods). The ejecta blanket alone then played the role of a gravity anomaly, leading to reorientation toward the tidal axis.

We used two different techniques to investigate the reorientation of Ganymede: the first technique balances the degree-2 gravity anomaly caused by a simplified ejecta blanket model against the remnant rotational bulge, and the second technique calculates the perturbation of the moment of inertia from a more complex ejecta blanket model without taking the remnant bulge into account.

### Evaluation on the basis of comparison with the remnant bulge

We evaluated the extent to which ejecta mass would have been sufficient to induce significant reorientation in Ganymede. Many significant studies have been conducted on the reorientation of solid bodies in the solar system^16–18,28–33^. For a rigid spinning tidally locked body, mass redistribution, such as cratering or subsurface diapirism, leads to a new principal axis rotational state (reorientation), while the remnant rotational and tidal bulge stabilizes the rotation axis. Therefore, the true polar-wander solutions are determined by the balance between the remnant bulge and a load (i.e., the mass creating the gravity anomaly)^34,35^. The dimensionless parameter $Q$, defined as the ratio of the degree-2 gravitational potential perturbation of the load to the fossil part of the rotational bulge, is useful in determining solutions^34,35^; this parameter is expressed as follows:

1 $$Q\equiv \frac{3\sqrt{5}GM{g}_{20}}{{\Omega }^{2}{R}^{3}\left({k}_{2}^{T*}-{k}_{2}^{T}\right)}$$

where ${g}_{20}$ is the degree-2 order-0 coefficient of the gravitational potential perturbation of the mass redistribution at the object surface; $G$ is the gravitational constant; $M$, $R$, and $\Omega$ are the mass, radius, and rotation angular frequency of Ganymede, respectively; and ${k}_{2}^{T*}$ and ${k}_{2}^{T}$ are the degree-2 fluid and actual Love numbers, respectively. If the load is too small compared with the existing bulge (i.e., $\left|Q\right|\ll 1$), reorientation does not occur because the bulge plays a role in stabilizing the current rotation axis. Here, $Q>0$ ($Q<0$) indicates that the load plays the role of a positive (negative) gravity anomaly, and $\left|Q\right|=1$ indicates that the gravity anomaly of the load is comparable with that of the rotational bulge. As an example, the load of $\left|Q\right|=1$ leads to a reorientation of 7° if its latitude is 45°^16^.

**Figure 2:**
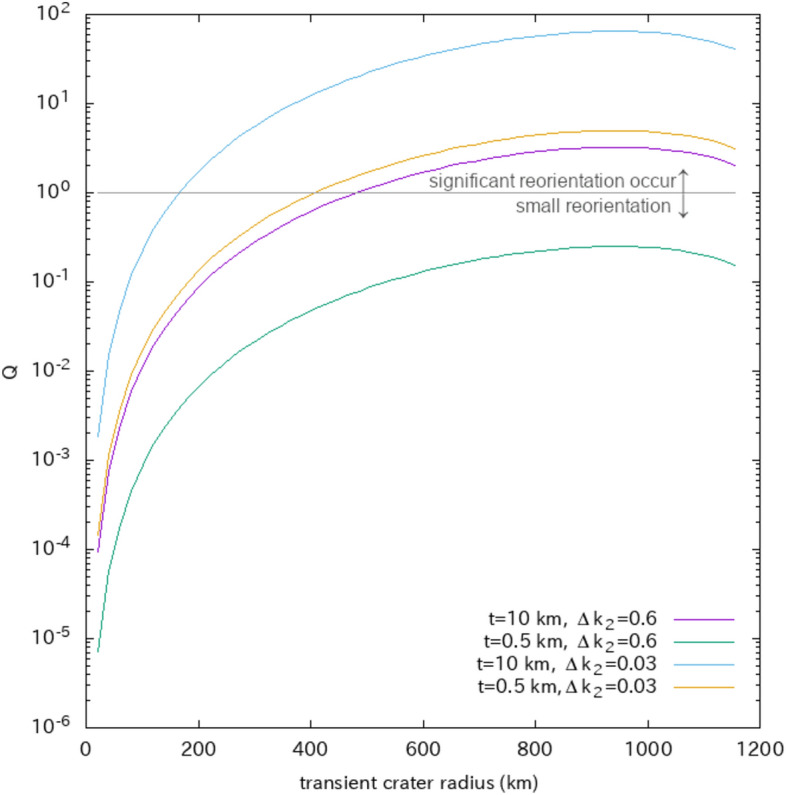
Parameter $Q$ is a function of the transient crater radius, where* t* represents the lithospheric thickness and where $\Delta {k}_{2}={k}_{2}^{T*}-{k}_{2}^{T}$ represents the difference between the degree-2 fluid and actual Love numbers. Here, $\left|Q\right|\gg 1$ ($\left|Q\right|\ll 1$) means that the load of the ejecta leads to significant (small) reorientation of Ganymede. Here, the gravity anomaly inside the basin radius is assumed to be zero.

If the ejecta blanket with a total volume of ${V}_{ejecta}$ has a uniform thickness and simple annulus shape with inner and outer radii of $R\phi$ and $R\theta$ ($\theta =2\phi )$ and isostasy is achieved within the basin radius ($R\phi )$, ${g}_{20}$ is given by.

2 $${g}_{20}=\frac{{\rho }_{c}(1-{C}_{n}){V}_{ejecta}}{2\sqrt{5}M}\left({\text{cos}}^{2}\phi +\text{cos}\phi \text{cos}\theta -{\text{sin}}^{2}\theta \right)$$

where $\phi$ and $\theta$ are the angles from the object center, ${C}_{n}$ is the degree of isostatic compensation, and ${\rho }_{c}$ is the density of the ejecta blanket (Methods). Note that this simple annulus provides a good approximation of the ejecta distribution because the wavelength of the degree-2 gravitational potential is sufficiently longer than the basin radius. On the basis of the Z model (e.g., ref.^36^), we assumed that the total volume ejected from a crater (${V}_{ejecta}$) was a fraction of the total volume displaced from the transient crater (${V}_{tc}$), ${V}_{ejecta}=\frac{1}{4}{V}_{tc}$, and that the size of the transient crater was a simple hemisphere, ${V}_{tc}=\frac{2}{3}\pi {{r}_{tc}}^{3}$. Additionally, we used a relation of $R\phi =1.3{r}_{tc}$ (e.g., ref.^36^). As a result, we obtain the value of $Q$ as a function of the transient crater radius, ${r}_{tc}$ (Fig. 2). The values of ${C}_{n}$ and $\Delta {k}_{2}={k}_{2}^{T*}-{k}_{2}^{T}$, which depend on the interior structure of Ganymede 4 billion years ago, are highly uncertain. Various estimations exist regarding the thickness of the lithosphere at the time of furrow formation: (i) the thickness of the lithosphere was estimated as 6–10 km on the basis of the width and spacing of furrows^37–39^, and (ii) the effective elastic thickness was estimated as 0.5 km on the basis of flexural uplift around furrows, although flexural uplift may have formed during a geologically active period of Ganymede long after furrow formation^40^. If the elastic thickness was $t=$ 10 km or 0.5 km, we can assume that ${C}_{n}$=0.87 or 0.99, respectively (Methods). When $t=$ 10 km, we can assume two cases: $\Delta {k}_{2}$=0.6 if Ganymede had no ocean and its mantle had a high viscosity of $\eta >$ 10^14^ Pa s or $\Delta {k}_{2}$=0.03 if Ganymede had an ocean or its mantle had a low viscosity of $\eta <$ 10^12^ Pa s (Methods). When $t=$ 0.5 km, we can assume two cases: $\Delta {k}_{2}$=0.6 if Ganymede had no ocean and its mantle had a high viscosity of $\eta >$ 10^14^ Pa s, or $\Delta {k}_{2}$=0.005 if Ganymede had an ocean or its mantle had a low viscosity of $\eta <$ 10^12^ Pa s (Methods). Figure 2 presents the four cases: $\left(t,\Delta {k}_{2}\right)$=(10 km, 0.6), (10 km, 0.03), (0.5 km, 0.6), and (0.5 km, 0.005). As a result, the volume of the ejecta blanket that creates a gravity anomaly with $Q=1$ corresponds to 100% of the ejecta created by a transient crater with radii of 480, 160, and 220 km when $\left(t,\Delta {k}_{2}\right)$=(10 km, 0.6), (10 km, 0.03), and (0.5 km, 0.005), respectively (Fig. 2). Therefore, a transient crater larger than 160 ~ 480 km in radius can lead to significant reorientation. When $\left(t,\Delta {k}_{2}\right)$= (0.5 km, 0.6), we can obtain $\left|Q\right|\ll 1$ for any transient crater radius.

### Global distribution of the ejecta blanket and the most stable orientation

The location of the Sputnik Planitia, which deviates 20° from the tidal axis, was explained by the balance between the bulge and mass anomaly of the basin^12^. However, the center of the furrow system cannot be explained in the same manner if the remnant bulge of Ganymede is readjusted on a short timescale. In general, some fraction of the remnant bulge eventually vanishes with the occurrence of reorientation; a new bulge is immediately formed in response to the new rotation state, and the gravity anomaly ultimately migrates to the tidal axis^15^. We calculated the center of the gravity anomaly (i.e., the minimum principal axis of the load) of the mass distribution of the ejecta blanket created by the furrow-forming impact. This approach ignores the stabilizing effect of any remnant bulge and would thus be expected to underestimate the size of the impactor compared with the results derived above for a simplified ejecta blanket. Numerical simulations and experimental studies have shown that although the shape of the crater depression is not sensitive to the impact incidence angle unless the angle is very shallow, the shape of the ejecta blanket is strongly sensitive to the impact incidence angle, and most impacts produce asymmetric ejecta patterns (Methods). Unless an ejecta blanket is perfectly symmetric, the center of the load does not match that of the crater. Therefore, we considered the impact incidence angle, utilized the methods described by Hirata et al.^41^ and Hirata^42^, calculated the trajectories of ejecta particles, and reproduced the thickness distribution of the ejecta blanket.

In this ejecta model, the initial launch position, velocity, and volume of ejecta particles were determined via the scaling law developed by Housen and Holsapple^43^ and Raducan et al.^44^. The ejecta model was obtained by experimental studies on Earth and has been used in studies of secondary craters and ejecta on icy satellites, including Ganymede^45,46^. Because the furrow-forming impact is in a gravity regime that does not depend on the strength of the target material, the interior structure of Ganymede would hardly affect the ejecta model. For example, an experimental study^47^ demonstrated that the shattering strength in impact experiments of icy clay samples with water contents of up to 35 wt% is not different from that of basalt, although the static tensile strength of basalt is approximately 8 times greater than that of icy clay samples. Housen and Holsapple^43^ provided three ejecta models in the gravity regime (water, dry sand, and glass microspheres as target surfaces), although the three ejecta models do not differ markedly from each other. The ejecta model of Housen and Holsapple^43^ was based on an experiment with a normal impact (incidence angle of 90°). Raducan et al.^44^ updated the ejecta model by including the incidence angle of the impactor as a parameter. The trajectories of the ejecta particles were solved via Hill’s equation^48^. We defined ejecta particles that reached below the object surface as colliding with the object and ejecta particles that reached an altitude greater than the Hill radius of the object as escaping from the object. Additionally, we removed the particles that land within the radius of a basin rim to assume an isostatically compensated basin floor.

**Figure 3:**
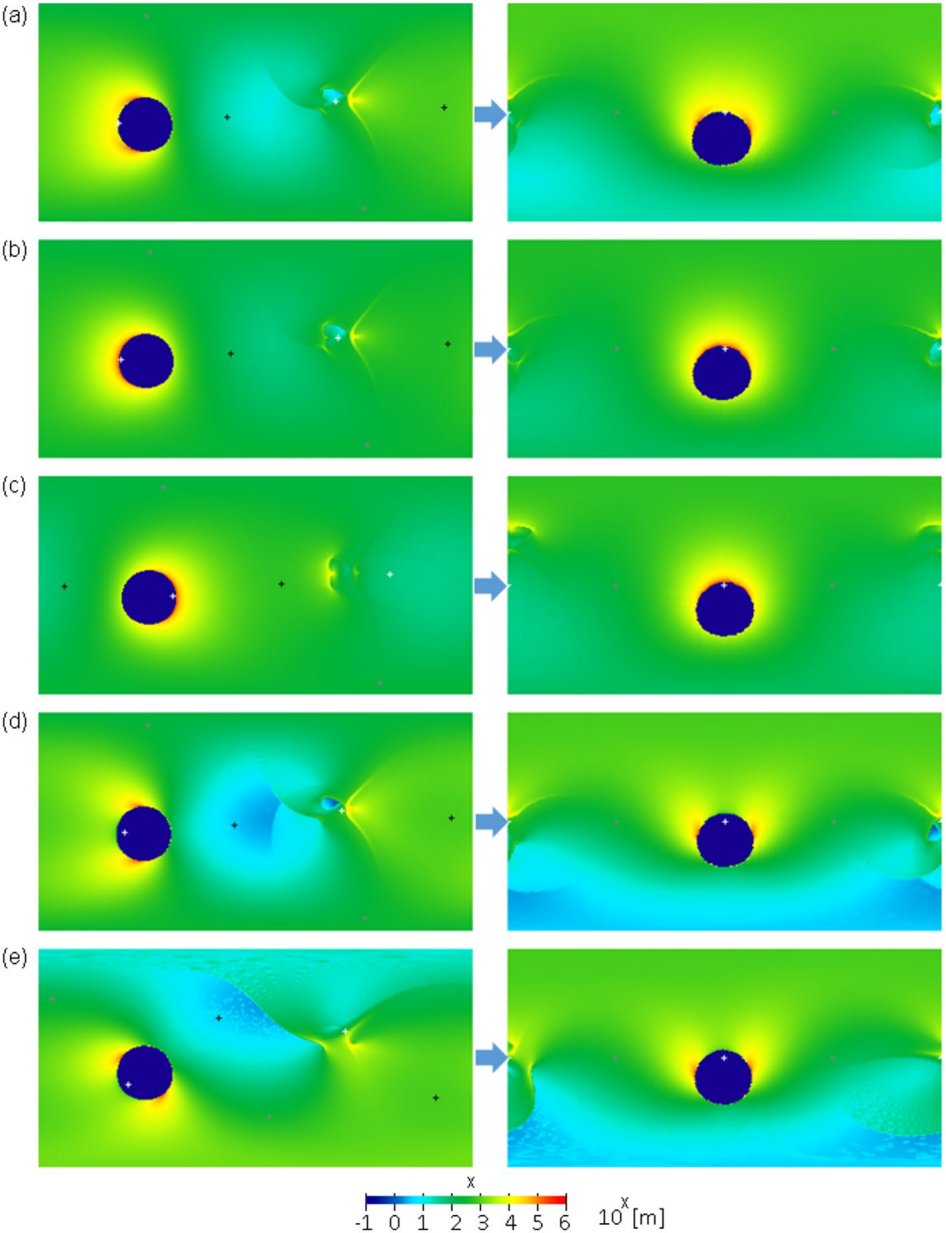
(left) Global distribution of ejecta blankets before reorientation using impact parameters #1–5 presented in Supplementary Table 1. The white, gray, and black “ + ” symbols represent the minimum, intermediate, and maximum principal axes, respectively. (right) Global ejecta thickness after reorientation to ensure that Ganymede had the most stable rotation. The vertical and horizontal axes in each plate indicate the latitude and east longitude, respectively, where the top left corner is 90° N and 0° E, and the bottom right corner is 90° S and 360° E. The color bar is represented on a logarithmic scale.

**Figure 4:**
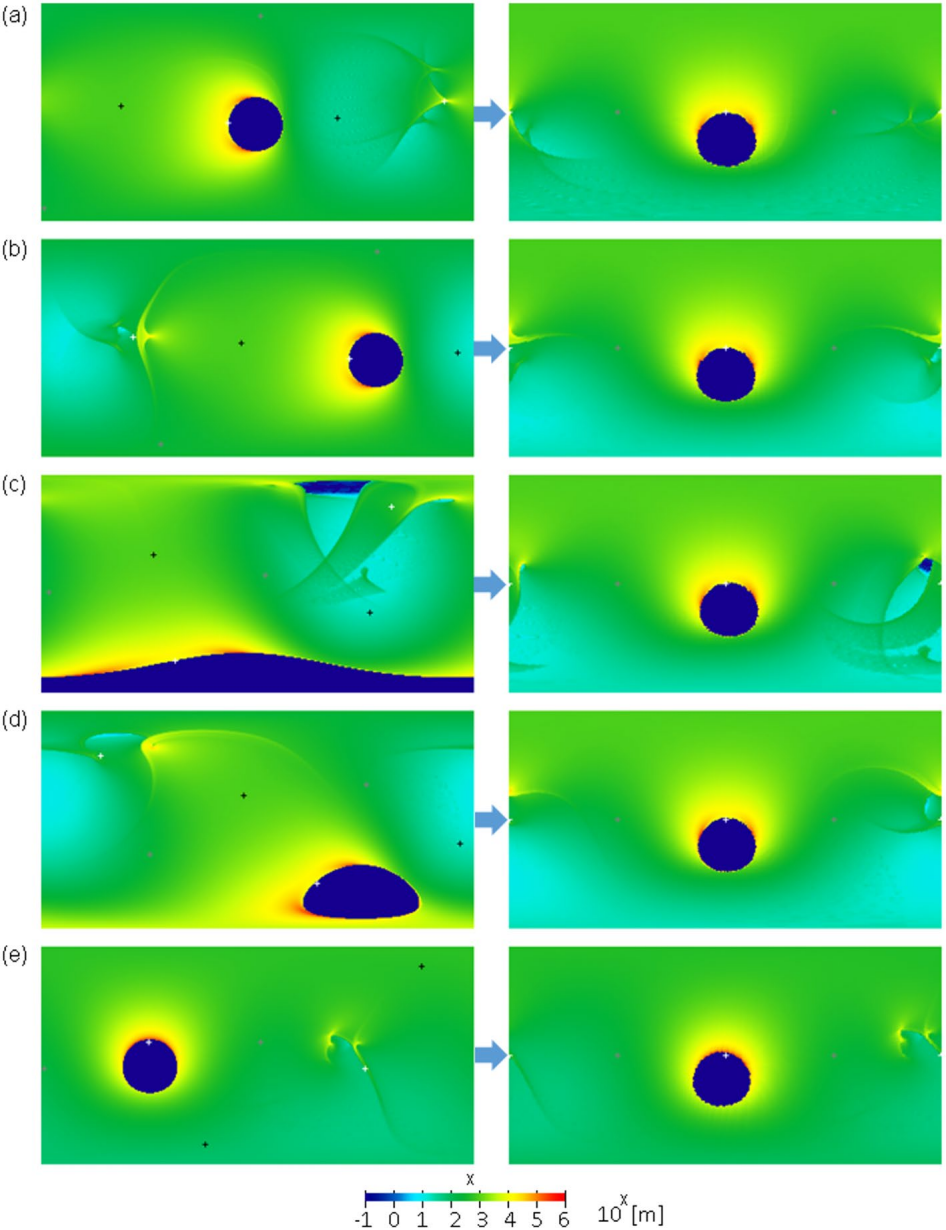
Global distribution of ejecta blankets before reorientation (left) and after reorientation (right) using impact parameters #6–10 presented in Supplementary Table 1. The white, gray, and black “ + ” symbols represent the minimum, intermediate, and maximum principal axes, respectively. The vertical and horizontal axes in each plate indicate the latitude and east longitude, respectively, where the top left corner is 90° N and 0° E, and the bottom right corner is 90° S and 360° E. The color bar is represented on a logarithmic scale.

**Figure 5:**
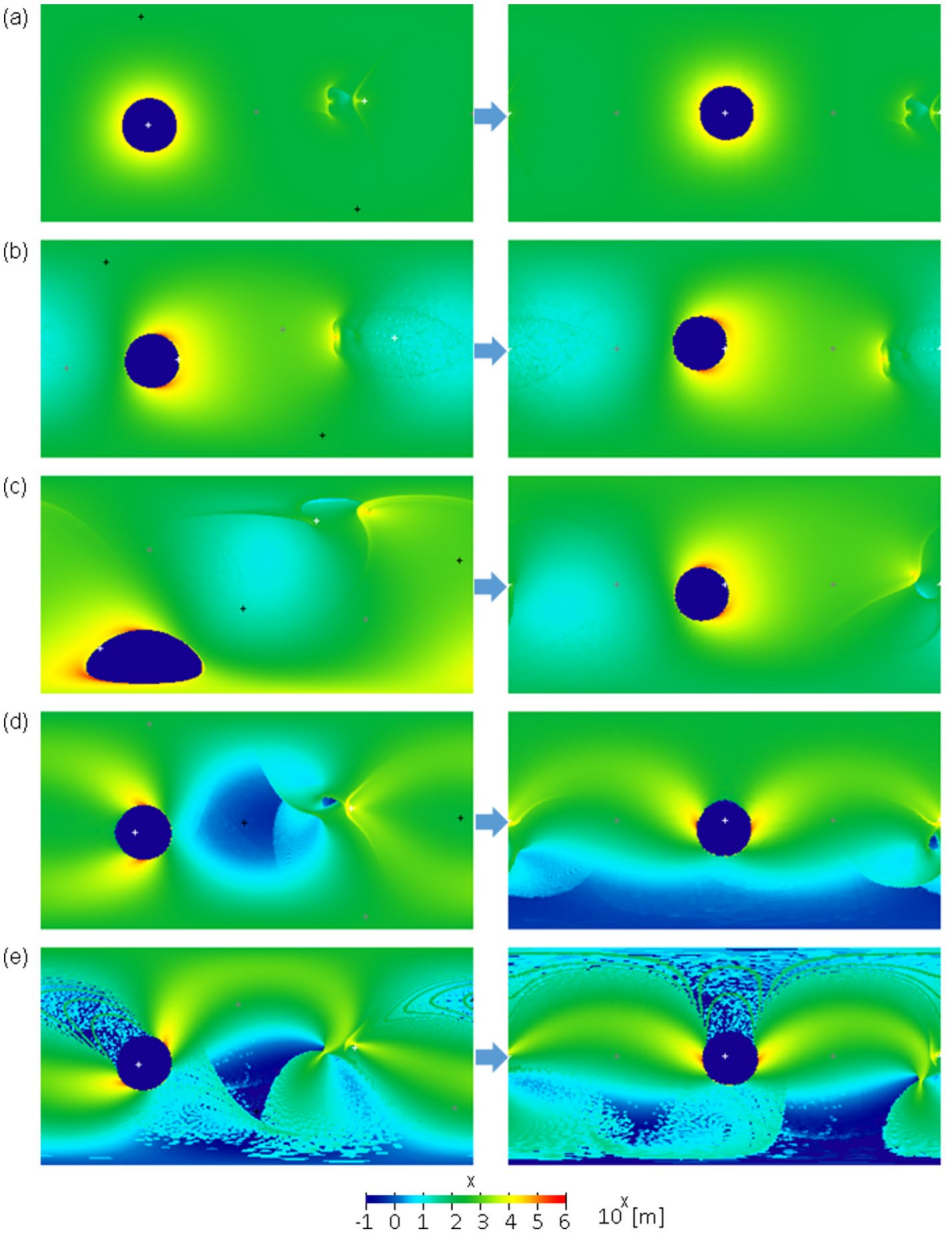
Global distribution of ejecta blankets before reorientation (left) and after reorientation (right) using impact parameters #11–15 presented in Supplementary Table 1.

The left plates in Figs. 3, 4, and 5 show fifteen examples of the global distribution of an ejecta blanket in the cases of an ejecta launch angle of 45°, C4 (dry sand as a target surface) as the set of scaling constants, a basin rim radius of $R\phi$=1000 km, and other parameters presented in Supplementary Table 1. We calculated the moment of inertia tensor of the mass distribution of each ejecta blanket and the eigenvalues and eigenvectors of the tensor to obtain the principal moments of inertia and principal axes of each ejecta blanket. We remapped each ejecta blanket in body-fixed coordinates after reorientation to ensure that Ganymede had the most stable rotation; the maximum and minimum principal axes of the mass distribution matched the rotation and tidal axes, respectively (right plates in Figs. 3, 4, and 5). In many cases, the current location of the center of the furrow system can be reproduced by this ejecta model (Figs. 3 and 4). However, this could not be reproduced in some cases (Fig. 5). Although reproducing the current location of the furrow system involves many parameters and is complicated, our results suggest that (i) the impact incidence angle is between 60° and 90° or (ii) the uprange direction is roughly on the west side. This is almost independent of the initial location of the impact site (Fig. 4).

In this ejecta model, a small mound with a height of approximately 10 km and a width of 300 ~ 600 km owing to the accumulation of ejecta appears near the sub-Jovian point (Figs. 3d, e, 4a, b). This may agree with the small mound with a height of 3 km located near the sub-Jovian point (1.5° E, 0.5° N) discovered by the Juno flyby and legacy data reanalysis^49,50^. When the uprange direction is roughly on the west side, the mound tends to appear near the sub-Jovian point after reorientation because the small mound shifted slightly from the exact opposite point of the impact owing to the effect of Ganymede's rotation. Viscous relaxation, isostatic readjustment, and/or uncertainty of the ejecta model may be responsible for the difference in height and shape between the discovered mound and our ejecta mound.

**Figure 6:**
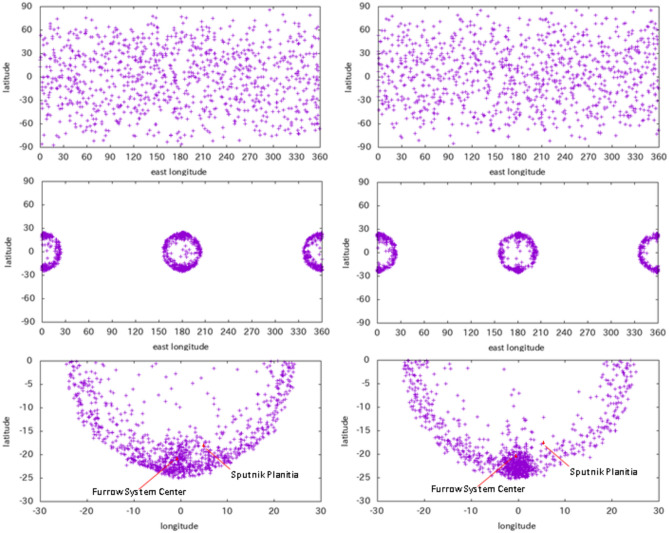
(top) Initial location of the center of the crater, (middle) final location of the center of the crater after reorientation, and (bottom) final location in the coordinate centered at the tidal axis. We have displayed cases with ejecta launch angles of 45° (left) and 30° (right). Note that because the true polar wander solution does not distinguish between the southern and northern hemispheres, points plotted in the Northern Hemisphere are shown with the opposite sign on the bottom plate. Owing to satellite rotation, it is not symmetrical with respect to the east‒west direction.

**Figure 7:**
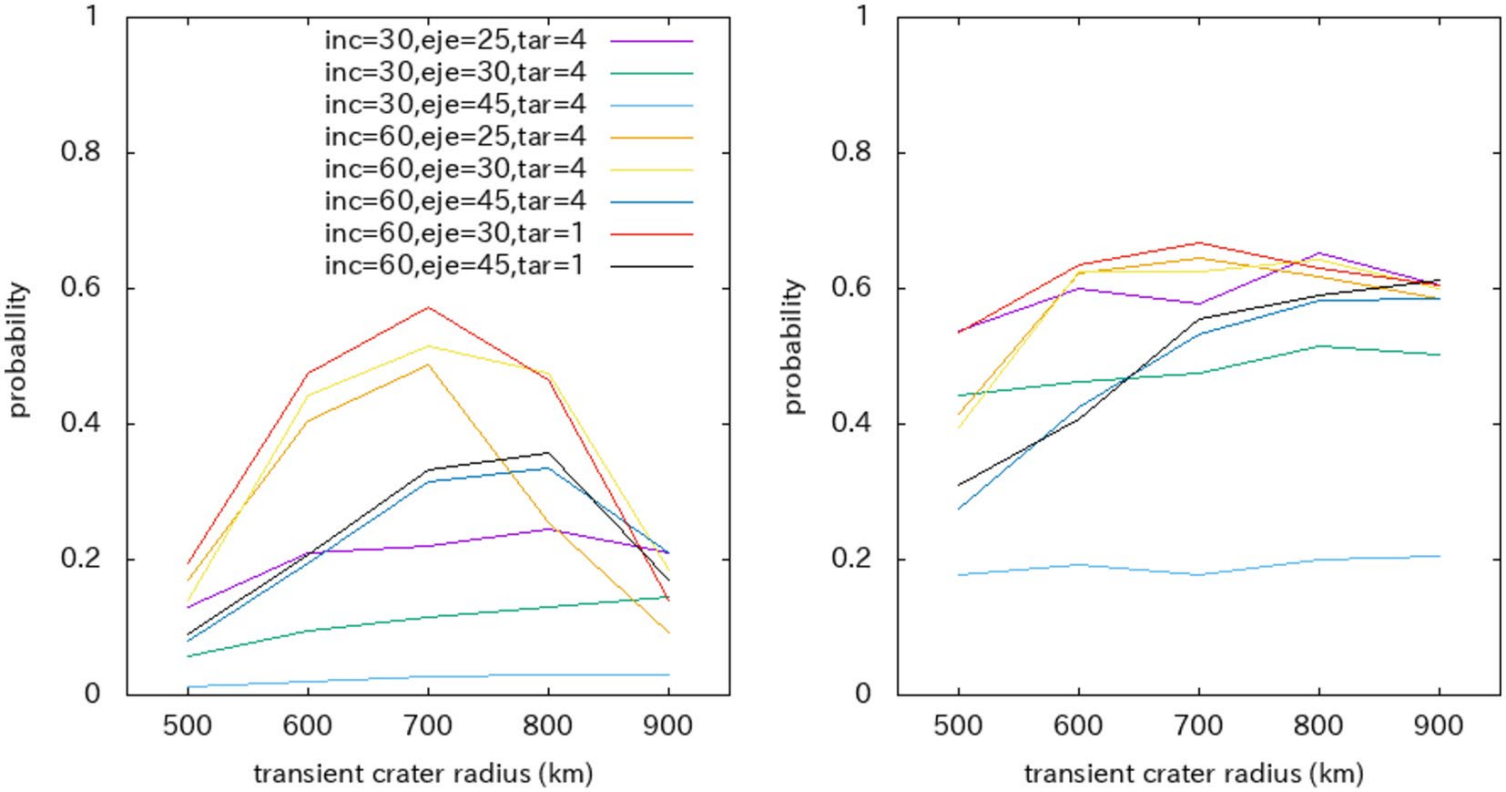
Probability that the center of the furrow system after the most stable reorientation moves within 5° (left) or 10° (right) from the point that deviates poleward by 20° from the tidal axis as a function of the transient crater radius. Here, we assume an impact incidence angle (inc) between 30° and 60° or between 60° and 90°; an ejecta launch angle (ej) of 25°, 30°, or 45°; and a target material (tar) of C1 or C4. The legend is common on both plates.

Furthermore, via the Monte Carlo method, we examined the probability that the center of the furrow system after the most stable reorientation matched the current location. The initial longitude, latitude, uprange direction, and incidence angle of the impactor follow the functions of $2\pi {x}_{1},\text{acos}\left(2{x}_{2}-1\right),2\pi {x}_{3}, \text{and}, \text{acos}(\sqrt{{x}_{4}})$, respectively, where $0\le {x}_{1},{x}_{2},{x}_{3},{x}_{4}<1$ are uniformly distributed random numbers^51^. We examined cases for an impact incidence angle between 60° and 90° or between 30° and 60°; a target material of C4 or C1 (water); a transient crater radius of 500, 600, 700, 800, or 900 km; and an ejecta launch angle of 25°, 30°, or 45° (angle between the initial launch velocity vector and the object surface). Note that an ejecta launch angle of ~ 28° may be the most appropriate for a giant impact because of the curvature of the target surface^52,53^. For each case, 1000 trials were performed. Figure 6 shows the initial and final locations of the crater centers in the case of an impact incidence angle between 60° and 90°, a target material of C4, and an ejecta launch angle of 30° or 45°. This figure shows that the basin center tends to shift poleward from the tidal axis by the basin radius along the tidal longitude. Figure 7 shows the relationship between the transient crater and the probability that the center of the furrow system after the most stable reorientation moved within 5° or 10° from the point deviated poleward by 20° from the tidal axis.

For example, the probability that the impact center falls within 5° from the point that deviates poleward by 20° from the tidal axis under the conditions of an impact incidence angle between 60° and 90°, a transient crater radius of 700 km, and an ejecta launch angle of 30° is 57.2% (Fig. 7). This transient crater radius creates a gravity anomaly with $Q>1$, as shown in Fig. 2. If reorientation does not occur, the probability is 0.76%. Note that there are four locations on Ganymede that deviate poleward by 20° from the tidal axis. The probability tends to be maximum when the size of the transient crater radius is 700 or 800 km, which is consistent with the above arguments for the *Q* value. A transient crater radius of 700 or 800 km is produced by an impactor with a radius of 150 km^11^. In conclusion, an impactor with a radius of 150 km and an incidence angle between 60° and 90° most likely reproduces the current location of the furrow system.

## Discussion

Although we demonstrated that the load of the ejecta blanket can reorient the impact site toward the tidal axis, a variety of other possible explanations for the reorientation of Ganymede cannot be ruled out because of the high uncertainty of the conditions of Ganymede 4 billion years ago. First, because inhomogeneous lithospheric thickness leads to an inhomogeneous degree of isostatic compensation, even if the ejecta blanket is perfectly symmetric, the ejecta rim supported by a thicker lithosphere would have greater gravity anomalies. Therefore, variations in lithospheric thickness, rather than an asymmetric ejecta distribution, may be responsible for the deviation of the center of the furrow system from the tidal axis by 20°. Second, it is proposed that the variation in the thickness of the icy shell driven by the latitudinal variation in solar radiation leads to the reorientation of Ganymede^32^ or that a captured rocky impactor core beneath the Sputnik Planitia basin leads to the reorientation of Pluto^33^, although we do not discuss these hypotheses in this paper. Third, the mass anomaly of the ejecta blanket and bulge supported by the lithosphere may vanish on a long timescale, whereas the presence of localized gravity anomalies (e.g., ref.^54^) indicates that relaxation does not always occur and that long-term mass anomalies can persist. However, it is difficult to estimate the relaxation time of the ejecta blanket and the remnant bulge (Method). If the gravity anomaly of the ejecta blanket vanishes before that of the remnant bulge, Ganymede should reorient back to its initial position. Alternatively, the cooling of Ganymede during the timeframe possibly increased the thickness of the lithosphere and should have fixed the orientation of Ganymede. These issues also need to be discussed in further studies.

The reorientation of Ganymede provides a window into the early history of the Jovian satellites. It is proposed that the 10-km-thick (0.5-km-thick) lithosphere of Ganymede indicates a surface heat flow of approximately 40 mW/m^2^ (60–80 mW/m^2^) at the time of furrow formation^39,40^, which exceeds the maximum plausible heat fluxes expected from radiogenic heating alone 4.5 billion years ago (nearly 27 mW/m^2^^55^). Therefore, additional energy sources, such as tidal heating, gravity segregation, and/or loss of accretional heat^40^, may have been present at the time of furrow formation. In particular, large-amplitude librations and/or nonsynchronous rotations can themselves cause significant heating due to tidal dissipation. We estimate that Ganymede should have attained its new orientation approximately 1000 years after the furrow-forming impact as a result of tidal dissipation (Methods). In addition, a 150 km-radius impactor was proposed to pump early Ganymede's free eccentricity^56^.

Heliocentric impactors preferentially hit the leading hemisphere of a tidally locked satellite, and the impact crater density of Ganymede decreases from the apex (center of the leading hemisphere) to the antapex (center of the trailing hemisphere) of its motion; however, the degree of apex–antapex asymmetry in various tidally locked satellites is considerably lower than that of theoretical estimates for ecliptic comets^51,57,58^. Previous studies^51,57,58^ have proposed various possible explanations for this difference, such as crater saturation, nonsynchronous rotation, reorientation, and nearly isotropic comets as the dominant impactor population. If the load of the ejecta has fixed the orientation of Ganymede, further reorientation would be unlikely in the case of Ganymede. This could be an area that needs further study.

The amount of ejecta in the Valhalla basin on Callisto is equivalent to $Q=$ 10, which is sufficiently large to cause a reorientation of Callisto (Methods); however, Valhalla is far from both the pole and tidal axes. Possible explanations include the following: (i) Callisto's rotation at the time of formation of the Valhalla basin was faster than that at present, (ii) isostasy is not archived on the basin floor, (iii) other surface loads exist on Callisto, or (iv) Callisto has a large remnant bulge; however, we cannot argue these explanations conclusively.

Many areas of Ganymede still have not been imaged with sufficient resolution^5^, and further data from future explorations are required for further discussions of the tectonic landforms formed by reorientation and the age of furrow formation. For example, tectonic patterns (other than furrows) resulting from Ganymede’s reorientation have not been discovered, although tectonic patterns resulting from the reorientation of Pluto and the Jovian satellites have been investigated^13,28,31^. New fractures may not have formed if the reactivation of existing fractures, or furrows, absorbed reorientation-induced stresses. The current accuracy of the gravity and topography measurements of Ganymede^59–61^ are insufficient for comparisons between individual topographic features owing to the lack of a global shape model of Ganymede and a local topographic map around the center of the furrow system. Although it is not clear that the gravity anomaly has vanished, the topographic profiles of the ejecta blankets should still be visible in the topography even if they are mostly viscously relaxed. Future explorations would reveal such a remnant of topographic profiles associated with the furrow-forming impact and the reorientation of Ganymede, which would provide insights into Ganymede’s early history and highlight its differences compared with those of other Jovian satellites.

## Methods

### The degree of isostatic compensation and the value of ${{\varvec{g}}}_{20}$

Following Melosh^19^, we assumed the following conditions for a crater basin: (i) the ejecta blanket of excavated material has a uniform thickness, ${t}_{e}$, and a simple annulus shape with inner and outer radii of $R\phi$ and $R\theta$, respectively, where $\phi$ and $\theta$ are the angles from the center of the object, respectively, and the outer radius is twice the inner radius, $\theta =2\phi$; and (ii) the basin floor has a uniform depth, $h$, and a simple circular shape with a radius of $R\phi$. In this case, a simple geometric solution gives the volume of the ejecta blanket and hole as follows:

3 $${V}_{ejecta}=2\pi {R}^{2}{t}_{e}(\text{cos}\phi -\text{cos}\theta ),\text{ and }$$

4 $${V}_{hole}=2\pi {R}^{2}h(1-\text{cos}\phi )$$

In general, both volumes are equal because of the conservation of mass^19^, even if the very shallow basin was created after the collapse of a transient crater; subsequently, we can use the simple relation of $h={t}_{e}(\text{cos}\phi -\text{cos}\theta )/(1-\text{cos}\phi )$. We considered the variations in the density of a nearly spherical object expressed by variations over a series of concentric spherical shells, and the density perturbations were azimuthally symmetric. The density perturbation in this model of the ejecta blanket model is expressed as follows:

5 $$\rho \left(r\right)-{\rho }_{0}\left(r\right)=\left\{\begin{array}{cc}\left\{\begin{array}{c}0 (\phi >\varphi >0)\\ {\rho }_{c} (\theta >\varphi >\phi )\\ 0 (2\pi >\varphi >\theta )\end{array} \right.& \left(R<r<R+{t}_{e}\right)\\ \left\{\begin{array}{c}-{\rho }_{c} (\phi >\varphi >0)\\ 0 (2\pi >\varphi >\phi )\end{array}\right.& \left(R-h<r<R\right)\\ 0& \left(r<R-h\right)\end{array}\right.$$

where ${\rho }_{0}\left(r\right)$ is the mean density for radius $r$, ${\rho }_{c}$ is the density of the crust, and $\varphi$ is the colatitude from the crater center. The degree-2 order-0 coefficient of the density perturbation is expressed as follows^62^:

6 $${\rho }_{20}\left(r\right)=\frac{\sqrt{5}}{4}{\int }_{0}^{\pi }\rho \left(r,\varphi \right)\left(3{\text{cos}}^{2}\varphi \text{sin}\varphi -\text{sin}\varphi \right) d\varphi$$

We can obtain.

7 $${\rho }_{20}\left(r\right)=\left\{\begin{array}{cc}\frac{\sqrt{5}}{4}{\rho }_{c}\left(\text{cos}\theta {\text{sin}}^{2}\theta -\text{cos}\phi {\text{sin}}^{2}\phi \right)& \left(R<r<R+{t}_{e}\right)\\ -\frac{\sqrt{5}}{4}{\rho }_{c}\text{cos}\phi {\text{sin}}^{2}\phi & \left(R-h<r<R\right)\\ 0& \left(r<R-h\right)\end{array}\right.$$

The degree-$l$ order-$m$ spherical harmonic coefficients of the gravitational potential at an arbitrary distance, $s (s\ge R)$, are given by^62^:

8 $${g}_{lm}(s)=\frac{4\pi }{M\left(2l+1\right){s}^{l}}{\int }_{0}^{R}{r}^{l+2}{\rho }_{lm}\left(r\right) dr$$

where $s$ is the distance from the center. Therefore, if this density perturbation is supported by an infinitely rigid lithosphere, the degree-2 order-0 gravitational potential coefficient at the object surface can be described as follows:

9 $${g}_{20}=\frac{\pi {R}^{2}{\rho }_{c}}{\sqrt{5}M}\left[{t}_{e}\text{cos}\theta {\text{sin}}^{2}\theta -\left(h+{t}_{e}\right)\text{cos}\phi {\text{sin}}^{2}\phi \right]$$

If we assume that $h={t}_{e}(\text{cos}\phi -\text{cos}\theta )/(1-\text{cos}\phi )$), this gravitational coefficient is always negative, ${g}_{20}<0$, which gives $Q<0$.

The actual shapes of the eject blankets in Fig. 3 are not simple annulus shapes. However, this simple annulus provides a good approximation of the ejecta distribution because the wavelength of the degree-2 gravitational potential is sufficiently longer than that of the basin radius and mostly averages inhomogeneity or asymmetry in the ejecta distribution. As a simple example, if we define $\theta =1.3\phi$ or $\theta =2.5\phi$ instead of $\theta =2\phi$, the value of $Q$ changes by only approximately 20–30%. Inhomogeneity in the tangential direction should have little effect on ${g}_{20}$ if the total mass of ejecta as a function from the center is equal to the simple annulus shape because Eq. (6) does not depend on the tangential direction.

A mass excess/deficit that is compensated for by isostasy does not create a free-air gravity anomaly, whereas the ejecta mass supported by the bending and membrane stresses of the lithosphere creates a gravity anomaly. Therefore, the gravity anomaly decreases by a factor of $(1-{C}_{n})$, where the degree of isostatic compensation, ${C}_{n}$, is described as follows^63^:

10 $${C}_{n}={\left[1-\frac{3{\rho }_{m}}{\left(2n+1\right)\overline{\rho }}\right]\left[\frac{\sigma \left({N}^{3}-4{N}^{2}\right)+\tau \left(N-2\right)+N-(1-\nu )}{N-(1-\nu )}-\frac{3{\rho }_{m}}{\left(2n+1\right)\overline{\rho }}\right]}^{-1}$$

where $N=n\left(n+1\right)$, $n$ is the degree of spherical harmonics of the horizontal width of the load ($n=2\pi R/w$, where $w$ is the horizontal width of the load),$\overline{\rho }$ is the mean density of the satellite, and $\tau$ and $\sigma$ are nondimensional parameters that are defined as follows:

11 $$\tau \equiv \frac{Et}{{R}^{2}g\left({\rho }_{m}-{\rho }_{c}\right)}\text{ and }\sigma \equiv \frac{\tau }{12\left(1-{\nu }^{2}\right)}{\left(\frac{t}{R}\right)}^{2}$$

where $t$ is the elastic thickness;${\rho }_{m}$ is the density of the mantle underlying the lithospheric plate; $E$ and $\nu$ are the Young’s modulus and Poisson’s ratio of the lithosphere, respectively; and $g$ is the surface gravity. Here, ${C}_{n}=0$ indicates that the load is fully supported by the lithosphere, and ${C}_{n}=1$ indicates that the load is fully compensated by isostasy. We assumed that $E$ = 9 GPa, $\nu$ =0.32 (ref. 64), ${\rho }_{m}$ = 1000 kg m^−3^, and ${\rho }_{c}$ = 930 kg m^−3^ (ref. 65). We assumed that $w=$ 1000 km because the ejecta blanket spreads across several basin radii. Note that the value of $w$ hardly affects ${C}_{n}$: ${C}_{n}=0.871$ ($w=$ 3000 km),${C}_{n}=0.875$ ($w=$ 1000 km), and ${C}_{n}=0.868$ ($w=$ 500 km) for $t=$ 10 km, and ${C}_{n}=0.992$ ($w=$ 3000 km),${C}_{n}=0.993$ ($w=$ 1000 km), and ${C}_{n}=0.993$ ($w=$ 500 km) for $t=$ 0.5 km. Consequently, we assume ${C}_{n}=0.87$ for $t=$ 10 km and ${C}_{n}=0.99$ for $t=$ 0.5 km.

If the value of ${C}_{n}$ is homogeneous inside and outside the basin, the gravity anomaly is always negative (${g}_{20}<0$); thus, reorientation that orients the basin center to the tidal axis does not occur. At least ${C}_{n}$ outside the basin must be smaller than ${C}_{n}$ inside the basin to obtain ${g}_{20}>0$. Nimmo et al.^12^ assumed that isostasy is archived inside the Sputnik Planitia basin. Similarly, in the case of Ganymede, the gravity anomaly becomes positive (${g}_{20}>0$) if (i) isostasy is archived inside the basin, which has the same attribute as $h=0$, and (ii) it is not archived outside the basin (i.e., the load of the ejecta is partially supported by the lithosphere). This assumption is natural for the following reasons. First, numerical simulations^26^ or impact experiments^66^ have proposed that the impactor can penetrate Europa’s ice shell and create conduits to the underlying ocean. Similarly, if Ganymede has an ocean and its ice shell is sufficiently thin, isostasy is easily achieved within the basin floor by the inflow of water. The furrow-forming impact alone may not create a large ocean because the size of a local melt pool created by the impact is up to 6 times the impactor radii^67^, which is less than 10% of the total volume of Ganymede. However, isostasy would be achieved within the basin floor even if Ganymede’s interior is warm convective ice without an ocean. Numerical simulations of warm convective ice without the ocean^11,27^ have shown that cold lithospheric material is replaced by warm subsurface material and that the postimpact lithospheric thickness becomes quite thin within the transient crater radius when the transient crater size is sufficiently larger than the lithospheric thickness. The lithospheric thickness recovered by cooling from the surface is roughly determined by the characteristic thermal diffusion depth, $\sqrt{\kappa {t}_{d}}$, where $\kappa$ is the thermal diffusivity and where ${t}_{d}$ is the timeframe^68^. The relaxation time of a load with a horizontal width of 100 km is 25 years (see below). If we use $\kappa =$ 2.9 $\times$ 10^–5^ for ice^69,70^ and ${t}_{d}=$ 25 years, we obtain $\sqrt{\kappa {t}_{d}}$= 150 m. An elastic thickness of $t=$ 150 m does not support a load above the lithosphere at all $({C}_{n}=1)$. In other words, the mass anomaly within the basin floor quickly disappears before the lithospheric thickness recovers. Therefore, isostasy can be achieved within the basin floor of the furrow system, regardless of the two surface models used. Second, numerical simulations^11,27^ have shown that the postimpact lithospheric thickness and temperature do not become thin and warm outside the basin. Numerical simulations for the Sputnik Planitia^71^ also show that hot preimpact thermal structures or cold thermal structures with an ocean greater than 150 km thick produce basins that are broadly isostatically compensated inside the basin and that are not fully isostatically compensated outside the basin. Therefore, it is likely that isostasy is not fully archived outside the basin. Third, furrow formation is unlikely to cause a significant change in the rheology of the lithosphere overall before and after the furrow-forming impact because individual furrows occur in the brittle part of the lithosphere^6,7^, and the brittle part above the brittle‒ductile transition layer should already include many cracks and faults due to impacts or tectonics. Notably, if the lithosphere is fully damaged by impact and isostasy is fully and broadly archived even outside the basin, any gravity anomalies will not be created by ejecta. If so, we should conclude that a coincidence or another hypothesis, such as the impactor-origin silicate mascon beneath the basin or the variation in the lithospheric thickness, is responsible for the current position of the center of the furrow system coinciding with the tidal axis.

Based on the above assumption ($h=0$), the dimensionless parameter becomes.

$$Q=\frac{3\pi G{\rho }_{c}(1-{C}_{n}){t}_{e}}{{\Omega }^{2}R\Delta {k}_{2}}\left[\text{cos}\theta {\text{sin}}^{2}\theta -\text{cos}\phi {\text{sin}}^{2}\phi \right]$$

or

12 $$Q=\frac{3G{\rho }_{c}(1-{C}_{n})}{{\Omega }^{2}R\Delta {k}_{2}}\frac{{V}_{ejecta}}{2{R}^{2}}\left({\text{cos}}^{2}\phi +\text{cos}\phi \text{cos}\theta -{\text{sin}}^{2}\theta \right)$$

The radius of the transient crater of the Gilgamesh basin is 135 km (ref. 46), which indicates a load of $Q$=0.027 ($\Delta {k}_{2}$=0.6) or 0.54 ($\Delta {k}_{2}$=0.03). The Gilgamesh basin does not cause significant reorientation of Ganymede, although a reorientation caused by the Gilgamesh basin has previously been proposed^28^. As another example, the mound at the sub-Jovian point, discovered by the Juno flyby and legacy data reanalysis, with a height of 3 km and an oval of 450 km × 750 km (refs. 49,50), has a value of $Q$=0.018 ($\Delta {k}_{2}$=0.6) or 0.36 ($\Delta {k}_{2}$=0.03), assuming $h$=-3 km, ${t}_{e}$= 0, $R\phi$=300 km, and ${C}_{n}=0$.87 (t = 10 km). Following Eq. (41) in Matsuyama and Nimmo^16^, the mound leads to very small reorientation of less than 2.6° if its latitude is 45°. We obtain a value of $Q$=0.17 for the mound if we assume that $h$=-3 km, ${t}_{e}$= 0, $R\phi$=300 km, ${C}_{n}=0.$ 99 (*t* = 0.5 km), and $\Delta {k}_{2}$=0.005.

### The degree 2 Love number of Ganymede

The value of $$\Delta {k}_{2}={k}_{2}^{T*}-{k}_{2}^{T}$$ is determined by the internal structure of Ganymede and has a significant effect on the value of $$Q$$. Many studies have investigated the tidal response of Ganymede, assuming various interior structure models^72–74^. For example, Moore and Schubert^72^ reported that $${k}_{2}^{T}$$ is approximately 0.01 if the present-day Ganymede has no ocean and its mantle has a high viscosity of $$\eta >$$ 10^14^ Pa s or approximately 0.4 to 0.6 if Ganymede has an ocean or its mantle has a low viscosity of $$\eta <$$ 10^12^ Pa s. Although it is unclear whether Ganymede’s interior is differentiated or undifferentiated, we estimate the Love number on the basis of a four-layer Ganymede model. Here, we assume that Ganymede consists of (1) a 10 km-thick elastic icy shell, (2) a viscoelastic icy mantle, (3) a viscoelastic rocky mantle, and (4) a liquid metallic core, where the rigidities of the icy shell and rocky mantle are 10 and 100 GPa, respectively; the densities of the icy shell, icy mantle, rocky mantle, and metallic core are 1050, 1050, 1050, 3100, and 5150 kg/m^3^, respectively; the outer radii of the icy shell, icy mantle, rocky mantle, and metallic core are 2638, 2628, 1745, and 710 km, respectively; and the viscosity of the rocky mantle is 10^20^ Pa s. The value of $${k}_{2}^{T*}$$ is defined as the value when the elastic icy shell is assumed to be a liquid ocean layer. Those rigidities, densities, outer radii, and viscosities are from Kamata et al.^73^. We use a code released by Kamata^75^ to calculate the periodic spheroidal deformation of a planetary body to obtain the Love number. As a result, when the viscosity of the icy mantle is 10^11^, 10^13^, or 10^15^ Pa s, we obtain ($${k}_{2}^{T},{k}_{2}^{T*})$$ = (0.579,0.604), (0.335,0.605), or (0.018,0.605), respectively. If the subsurface ocean layer (with a density of 1050 kg/m^3^) is present instead of the viscoelastic icy mantle, we can obtain ($${k}_{2}^{T},{k}_{2}^{T*})$$ =(0.579,0.604). Considering the large uncertainties, we studied the two cases of $$\Delta {k}_{2}$$=0.03 and $$\Delta {k}_{2}$$=0.6 for *t* = 10 km. If we assume a 0.5 km-thick elastic icy shell instead of a 10 km-thick elastic icy shell, when the viscosity of the icy mantle is 10^11^, 10^13^, or 10^15^ Pa s, we obtain ($${k}_{2}^{T},{k}_{2}^{T*})$$ =(0.599,0.604), (0.446, 0.605), or (0.018,0.605); therefore, we study the two cases of $$\Delta {k}_{2}$$=0.005 and $$\Delta {k}_{2}$$=0.6 for* t* = 0.5 km. This combination of $$\Delta {k}_{2}$$=0.6 and* t* = 0.5 km may be unlikely because the very thin lithosphere and cold interior are not consistent.

### Tidal decay of rotation

Following a large impact event, tidal friction quickly dampens the motions of the satellite’s orientation, such as librational oscillations and nonsynchronous rotation, and the satellite attains its new orientation on a short timescale^19^. The timescales of the tidal decay of librational rotation and nonsynchronous rotation have been estimated in previous studies^19,76,77^, and all three studies were equivalent. Following Noyelles et al.^77^, the timescale is expressed as follows:

13 $${T}_{damp}=\frac{2}{3}\frac{{Q}_{d}}{{k}_{2}^{T}}\frac{GC}{{{n}_{o}}^{3}{R}^{5}},$$

where $C$ is the maximum moment of inertia about the center of mass; ${n}_{o}$ is the mean orbital motion; and ${Q}_{d}$ is the specific dissipation factor of the satellite. Assuming a homogenous interior ($C=0.4 M{R}^{2}$) and reasonable values for warm icy satellites,${k}_{2}^{T}$=0.5 and ${Q}_{d}$=100 (ref. 78), we can obtain ${T}_{damp}$=870 years. If Ganymede was cold and rigid (${Q}_{d}/{k}^T_{2}$ ~ 10^5^), we can obtain ${T}_{damp}$=4.4 million years.

### Viscous relaxation of the load and bulge

The mass anomaly not supported by the lithosphere disappears owing to viscous relaxation on a timescale of $\eta /{\rho }_{m}gw$ (ref. 68), where $w$ is the horizontal width of the load. The icy materials underlying the elastic layer are convecting, and their reference viscosity is as low as $\eta$= 10^12^ to 10^17^ Pa s (ref. 79). For example, a 100 km-wide load not supported by the lithosphere would vanish in less than 25 years. Similarly, the density perturbations below the lithosphere created by heating and redistribution of the impact crater would vanish on a short timescale. If the relaxation time of the remnant tidal/rotational bulge is given by $\eta /{\rho }_{m}gR$ (ref. 17), the bulge would vanish in 1 year. However, this estimate is suitable only for an isoviscous case, whereas the internal structure of Ganymede is much more complicated and, in particular, may have a rigid outer shell that can maintain a remnant bulge over geological time. In short, the actual bulge relaxation timescale is extremely uncertain. It is estimated that old craters on the dark terrain of Ganymede have relaxed viscously for 1 billion years^55,80^. The timescale of relaxation of the mass anomaly of the ejecta blankets and the tidal/rotational bulge may also be approximately 1 billion years.

### The Valhalla basin

The original crater radius of the Valhalla basin was estimated to be 500 km on the basis of the mapping of ejecta and secondaries^81^. The thickness of the lithosphere was estimated to range from 15 to 20 km during the formation of the Valhalla basin^6^. Assuming $R\phi$=500 km, $t=$ 20 km,$h=$ 0, $w$= 500 km, $n$= 15, ${C}_{n}$=0.66, and physical parameters for Callisto, we can obtain $Q$=199.8 ($\Delta {k}_{2}$=0.03) or $Q$=10.0 ($\Delta {k}_{2}$=0.6) for the Valhalla basin, which should lead to a significant reorientation of Callisto. Possible explanations for this include Callisto's rotational period, incomplete isostatic compensation within the basin floor, and other surface loads. If Callisto's rotational period was 50 h (currently 400 h), we could obtain $Q$=0.16 ($\Delta {k}_{2}$=0.6). The Valhalla basin is one of the oldest surface features on Callisto^21,82^, and Callisto at the time of the formation of the Valhalla basin may not have yet reached a synchronous rotation state. However, this scenario may be unlikely because Callisto attains a synchronous rotation state in 2 million years if we use ${Q}_{d}/{k}_{2}$ ~ 20 (ref. 83). If Callisto was cold and rigid (${Q}_{d}/{k}_{2}$ ~ 10^5^), Callisto obtained a synchronous rotation state 1 billion years after its formation. Note that because Ganymede attains a synchronous rotation state at 0.66 million years for ${Q}_{d}$=100 after its formation^76^, Ganymede’s rotational period at the time of furrow formation should not differ much from the present period. If isostasy is not achieved within the basin floor of the Valhalla basin (i.e., $h>0$), incomplete isostatic compensation of the negative topographic profile within the basin would decrease $Q$. Finally, as many areas of Callisto have not yet been imaged with sufficient resolution, we cannot rule out the possibility that large undiscovered loads exist on Callisto’s surface. Additionally, Callisto may have had a large remnant bulge, such as the equatorial bulge of Iapetus, because of Callisto’s undifferentiated and inert interior.

### Note for the calculation of the ejecta blanket

Many studies have focused on the shape of the ejecta blanket, which shows that the mass distribution of ejecta is strongly sensitive to the impact incidence angle and highly asymmetric^84–88^. Therefore, most naturally, the impact incidence angle is responsible for the asymmetric load to explain the location of the center of the furrow system. However, there could be various alternative explanations, as we described above.

It is not unnatural that a thick ejecta blanket would be superimposed on where the furrows lie because this is also the case in the Valhalla basin. The Valhalla basin can be divided into three distinct zones outward from the center: a central smooth zone (*r*_*b*_ < 360 km, where *r*_*b*_ is the distance from the basin center), an inner ridge and trough zone (360 km < *r*_*b*_ < 950 km), and an outer graben zone (950 km < *r*_*b*_ < 1900 km). The crater density in the central smooth zone and inner ridge and trough zone is 3.5 times lower than that in the adjacent unmodified cratered terrain, whereas the crater density in the outer graben zone is intermediate between that in the inner zones and unmodified cratered terrain and apparently increases linearly outward; moreover, the deficiency of craters is interpreted to be due to obliteration by continuous ejecta blankets^3,21^. If troughs and grabens were created first, they would be damaged by ejecta particles. Therefore, troughs and grabens were likely created after the accumulation of ejecta. Similarly, no structures older than the furrows have been found on the Ganymede surface, and no craters cut by the furrows have been found^3^. Therefore, all craters on Ganymede were likely created after furrow formation (perhaps, all ancient craters created before furrow formation were removed by ejecta). Compared with ejecta particles flying in a ballistic trajectory, the timing of furrow formation formed by the asthenospheric flow would be delayed.

The thickness of the ejecta blankets at the rim is 100 km in our calculation; however, this is unrealistic. This is mainly because our ejecta model does not consider any subsequent movement after the landing of ejecta particles, which creates a point of discontinuity in the thickness along the basin rim. In reality, some dispersion induced by mass wasting or re-ejection should occur. The ejecta volume at a point of discontinuity is not large; thus, we expect that it hardly affects our results. Nevertheless, we recalculated the ejecta distribution in the vicinity of the basin rim, assuming a realistic surface slope. Specifically, we removed ejecta steeper than a given slope ($\uplambda$) around the basin rim, calculated the moment of inertia tensor of the mass distribution of the ejecta blanket whose steep part was removed, and remapped the ejecta blanket after the most stable reorientation (Supplementary Fig. 1). Here, we assume that the removed ejecta do not create a mass anomaly. Although this calculation does not assume a particular geologic process, it is known that a portion of the ejecta rim moves into the basin floor due to lateral movement, such as mass wasting. Thus, the ejecta mass within the basin does not create a free-air gravity anomaly because we assume that isostasy is archived inside the basin. The 15 examples in this figure correspond to the 15 examples shown on the right side of Figs. 3, 4, and 5. As a result, among the 15 examples, only #3 for $\uplambda \le$ 20 degrees is different from the original example. For the rest, we find that almost the same principal axis rotation state is obtained. It is unclear how much gradient is actually allowed on Ganymede’s surface; however, this figure shows that the discontinuities around the basin rim hardly affect our results. This is mainly because the unrealistically thick ejecta rim is created by the accumulation of ejecta in a very small area, and the removed ejecta mass was approximately less than 10% of the total mass when $\uplambda \ge$ 20 degrees and less than 20% when $\uplambda =$ 5 degrees.

We performed similar numerical simulations for Pluto. The simulations are almost the same as those for Ganymede, other than the trajectories of the ejecta particles. The trajectories were solved via the equation of motion for the circular restricted three-body motion (Pluto, Charon, and an ejecta particle)^48^. Supplementary Fig. 2 shows a similar probability as a function of the transient crater radius, similar to that in Fig. 7. The mean radius of the Sputnik Planitia is 550 km (ref. 13), which corresponds to a transient crater radius of 420 km. For this transient crater radius, the probability that the impact center is within 5° from the point that deviates poleward by 20° from the tidal axis is approximately 20–30%. Therefore, the center of the Sputnik Planitia of Pluto, which, together with the furrow system, deviates poleward by 20° from the tidal axis, can be explained by a similar model.

## Supplementary Information

Supplementary Information.

## Data Availability

The software for calculating ejecta blankets is available at GitHub (https://github.com/naoyukihirata/ganymede-ejecta).
