# Supplementary material for: Giant impact on early Ganymede and its subsequent reorientation
Source: Sci Rep. 2024 Sep 3;14:19982. doi: 10.1038/s41598-024-69914-2 (PMC11371838; doi:10.1038/s41598-024-69914-2)
Supplement: Supplementary file 1 — Supplementary Information. [file 41598_2024_69914_MOESM1_ESM.docx]

**Supplementary Table 1.** Parameters for each ejecta blanket

| # | Fig. | Lon.^*1^ | Lat.^*1^ | uprange direction ^*2^ | incidence angle ^*3^ |
| --- | --- | --- | --- | --- | --- |
| 1 | 3a | 90 | -10 | 0 | 70 |
| 2 | 3b | 90 | -10 | 0 | 80 |
| 3 | 3c | 90 | -10 | 180 | 80 |
| 4 | 3d | 90 | -10 | 0 | 60 |
| 5 | 3e | 90 | -10 | 45 | 60 |
| 6 | 4a | 180 | -10 | 0 | 70 |
| 7 | 4b | 280 | -10 | 0 | 70 |
| 8 | 4c | 180 | -80 | 0 | 70 |
| 9 | 4d | 270 | -60 | 0 | 70 |
| 10 | 4e | 90 | -10 | 270 | 80 |
| 11 | 5a | 90 | -10 | 0 | 90 |
| 12 | 5b | 90 | -10 | 180 | 70 |
| 13 | 5c | 90 | -60 | 0 | 70 |
| 14 | 5d | 90 | -10 | 0 | 50 |
| 15 | 5e | 90 | -10 | 315 | 30 |

*1 East longitude and latitude of the impact site before reorientation.

*2 Uprange direction in degrees, anticlockwise from east.

*3 Incidence angle of the impactor from the surface.


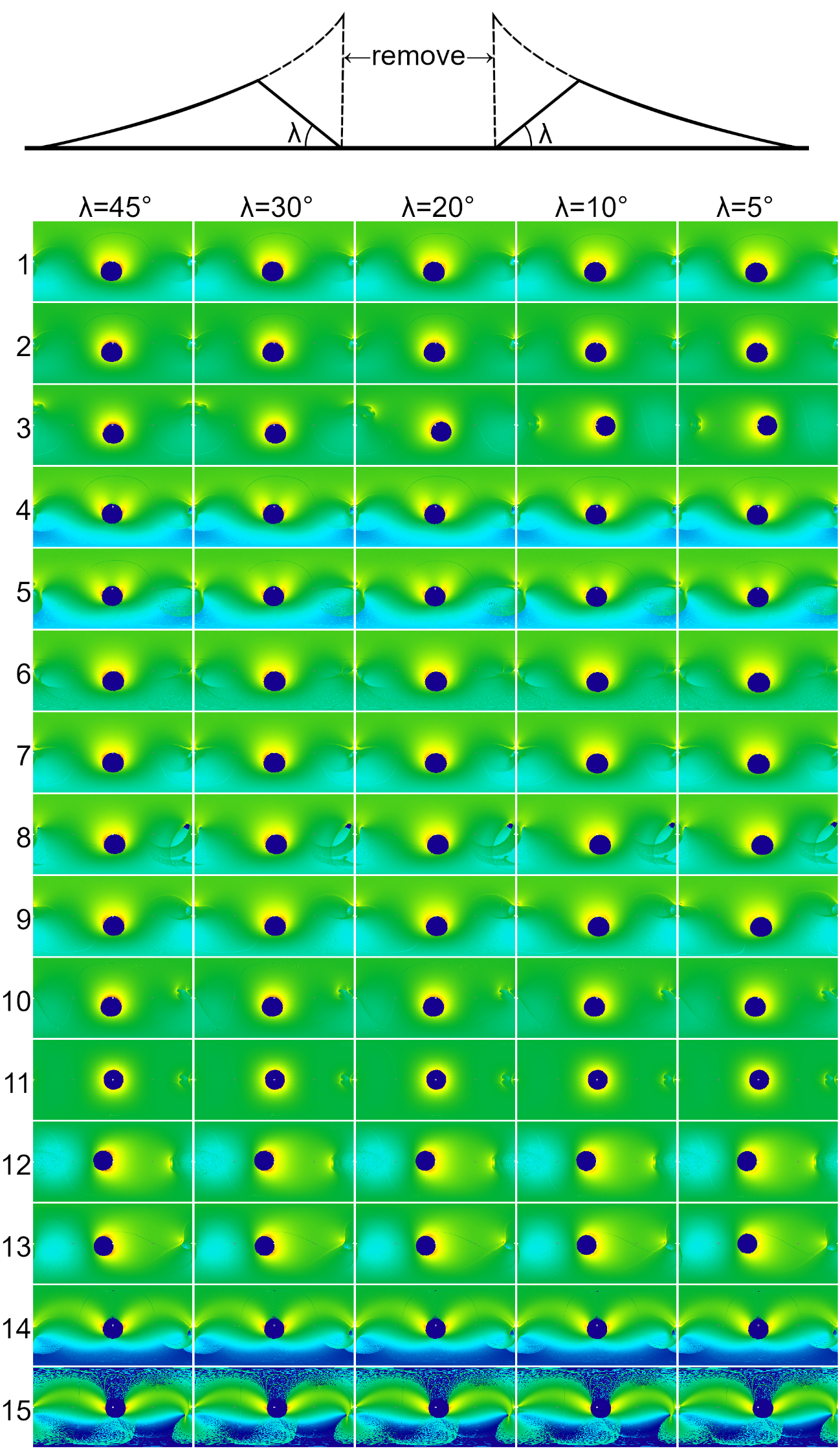


**Supplementary Figure 1.** Ejecta distribution after the most stable reorientation, assuming an ejecta blanket whose steep part (>λ) was removed. The 15 examples (indicated by the left column numbers) correspond to the 15 examples of Figs. 3, 4, and 5 and Supplementary Table 1. Here, we assume that λ=45, 30, 20, 10, and 5 degrees (from left to right). The color scale bar is similar to that in Figs. 3, 4, and 5.

**
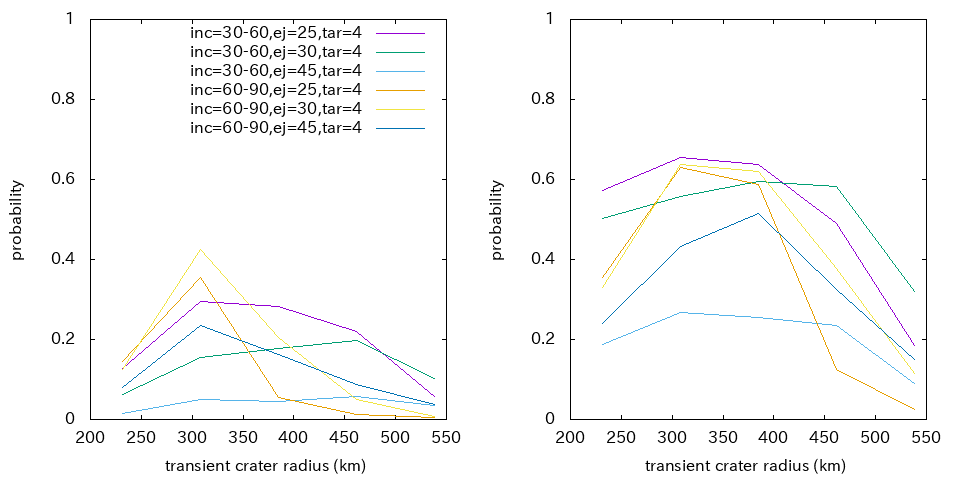
**

**Supplementary Figure 2.** Probability that the center of the Sputnik Planitia after the most stable reorientation moves within (left) 5° or (right) 10° from the point that deviates poleward by 20° from the tidal axis as a function of the transient crater radius, assuming an impact incidence angle (inc) between 30° and 60° or between 60° and 90°; an ejecta launch angle (ej) of 25°, 30°, or 45°; and a target material (tar) of C4.
